# Mapping the lack of public initiative against female genital mutilation in Denmark

**DOI:** 10.1186/s12978-018-0499-2

**Published:** 2018-04-07

**Authors:** Gro Møller Christoffersen, Peter James Bruhn, Rosanna de Neergaard, Susanne Engel, Vibeke Naeser

**Affiliations:** 1 University of Copenhagen, Faculty of Health and Medical Sciences, Blegdamsvej 3, 2200 Copenhagen N, Denmark; 2 Department of Surgical Gastroenterology, Copenhagen University Hospital North Zealand, Dyrehavevej 29, 3400 Hillerød, Denmark; 3 Department of Emergency Medicine, Zealand University Hospital Slagelse, Ingemannsvej 50, 4200 Slagelse, Denmark; 4 Department of Culture, Municipality of Sermersooq, Kuussuaq 2, 3900 Nuuk, Greenland; 5 Department of Obstetrics and Gynecology, Zealand University Hospital Næstved, Ringstedgade 61, 4700 Næstved, Denmark

**Keywords:** Female genital mutilation, Female genital cutting, Migrant health, Pricking

## Abstract

**Background:**

Female genital mutilation (FGM) is a harmful practice prevalent in 35 countries, mainly in Africa, as well as in some Middle Eastern countries and a few Asian countries. FGM comprises all procedures that involve partial or complete resection of, or other injury to, external female genitalia for non-medical reasons. The practice of FGM has spread to Western countries due to migration. The European Institute for Gender Equality recommend that FGM be combatted by nationally coordinated efforts through implementation of national action plans, guidelines for professionals as well as comprehensive research in the field. FGM was outlawed in Denmark 2003, but no national actions plan has been implemented. Instead, the task of combatting FGM is currently under the responsibility of local governments in the form of the 98 municipalities. The aim of this study is to investigate the Danish municipalities’ efforts to prevent FGM on the local level, and whether these initiatives are in accordance with international recommendations and standards.

**Methods:**

All 98 Danish municipalities were invited to respond to a questionnaire regarding FGM in their respective municipalities. The inclusion process and questionnaire was designed after a pilot study, which included 29 municipalities. The questionnaire consisted of four overall areas of focus: “action plan”, “registration”, “information material” and “preventive initiatives”. Demographic data were gathered from the 2017 census by Statistics Denmark. Risk countries were defined as countries with a tradition for FGM, identified from the 2016 UNICEF definition.

**Results:**

A total of 67 municipalities participated in the study. At the time of census, 1.8% of the Danish population was immigrants with origins in risk countries. A total of 10.4% of the responding municipalities indicated to have implemented a specific action plan against FGM. A total of 7,5% had implemented specific preventive initiatives against FGM. Registration of reported FGM cases were indicated to be performed in 73.1% of the responding municipalities; however, only 17.9% stated to perform registration of FGM specifically as such, and not as general child abuse.

**Conclusions:**

Our study shows that the current situation of FGM registration and prevention being under local administrative responsibility in the 98 Danish municipalities has led to a severe lack of coordinated public initiative against FGM.

**Electronic supplementary material:**

The online version of this article (10.1186/s12978-018-0499-2) contains supplementary material, which is available to authorized users.

## Plain English summary

Female genital mutilation (FGM) is a harmful practice where female external genitalia are injured or partly or completely removed due to non-medical reasons. The practice of FGM has spread to European countries with immigration from countries with a tradition for FGM. The European Institute for Gender Equality recommends that FGM should be combatted by nationally coordinated preventive efforts in the form of, among other things, a national action plan. FGM was outlawed in Denmark in 2003, but no national action plan has been implemented. The task of combatting FGM is currently under the responsibility of local governments in the form of the 98 municipalities. We sought to investigate whether FGM is combatted in accordance with international standards on the local level. We invited the Danish municipalities to respond to a questionnaire regarding FGM prevention in their respective municipalities. We also analyzed demographic data regarding distribution of immigrants from countries that practice FGM, across the respective municipalities. We found that only very few Danish municipalities have implemented specific action plans against FGM, or have implemented specific preventive initiatives against FGM. Overall, only very few Danish municipalities live up to international recommendations and standards regarding FGM prevention. Thus, we conclude that the current situation of FGM registration and prevention being under local administrative responsibility in the 98 municipalities has led to a severe lack of coordinated public initiative against FGM in Denmark.

## Background

Female genital mutilation (FGM) is a harmful practice prevalent in 35 countries, mainly in Africa, as well as in some Middle Eastern countries and a few Asian countries [1]. The practice is described by the World Health Organization (WHO) as follows: “Female genital mutilation (FGM) comprises all procedures that involve partial or total removal of the external female genitalia, or other injury to the female genital organs for non-medical reasons (…) FGM is recognized internationally as a human rights violation. It reflects deep-rooted inequality between the sexes, and constitutes an extreme form of discrimination against women” [2]. Due to migration over the past decades the practice of FGM has spread to European and other Western countries [3–10]. It has been estimated by the European Parliament that 500,000 women and girls have been subjected to FGM whereas 180,000 girls are in risk of being subjected to FGM [11]; though some argue that this is an under-estimation [4].

Female genital mutilation was outlawed in Denmark in 2003. In 2013, the European Institute for Gender Equality (EIGE) published an official recommendation for Denmark to define and implement a national action plan such as “national guidelines for professionals across a variety of sectors as well as coordination of the effort to combat FGM. Comprehensive and in-depth research in the field is also needed as well as prevalence surveys of FGM in Denmark” [12]. However, several changing governments have not implemented a national action plan to prevent and combat FGM in Denmark. A proposal for a national action plan was drafted by Danish NGOs and a number of individual authors in 2007–2008 and was submitted to the Health Committee of the Parliament in 2009. This action plan was not implemented. Instead, the 98 Danish municipalities individually administrate the responsibility of combatting FGM on a local level.

The lack of political prioritization of the FGM issue in Denmark is unusual in a Nordic context. Finland, Sweden and Norway, along with several other European countries, have implemented comprehensive national and international guidelines to combat FGM [12–17].

In this study, our aim is to investigate the Danish municipalities’ initiatives to prevent FGM on the local level, and whether these initiatives are in accordance with international recommendations and standards.

## Methods

### Questionnaire

In order to gain insight into if and how FGM is combated through public efforts on the local level in the 98 Danish municipalities, a questionnaire was developed. We designed the entirety of the questionnaire, since a validated questionnaire on the subject of FGM prevention on the local governmental level has not yet been published. During the questionnaire design process, we used a checklist proposed by Eysenbach et al. [18]. The questionnaire consisted of four main areas of focus, which we defined as “action plan”, “registration of FGM”, “information material” and preventive initiatives. These areas of focus were chosen in accordance with the concluding focus points described by EIGE in their 2013 report on the subject of FGM in Denmark, as well as efforts described in other Scandinavian action plans regarding FGM [12, 13, 16]. In those parts of the questionnaire where doubt about terminology could have an impact on answers, the question was supplemented with a definition, so as to ensure measurement validity. An action plan was defined as “A point to point guideline for professionals on how to act when being made aware of a case of FGM or of a girl in potential risk of FGM”. Most questions were closed-ended with possible answers being yes/no/do not know. When questions were not close-ended, these could be answered with different given categories or free text (Additional file 1). The questionnaire was answered through a webpage and took few minutes to complete.

### Pilot project

A total of 29 municipalities in the Capital Region in Denmark were invited to participate in a pilot project, which consisted of the questionnaire described earlier. The specific department in each of the municipalities with the main responsibility for children, reports of child abuse or likewise, were identified through the respective municipality’s website or by telephone. By contacting the general manager with the overall responsibility for the department in question within each municipality, contact information for the individual employee appointed in charge of handling reports of cases of FGM, and with the most adequate knowledge about the particular area, was acquired. The questionnaire was then sent electronically to this person as the potential responder. If the initial contact did not result in a reply, the municipalities were then contacted by telephone or by email once more. The questionnaire was then revised, taking into account the feedback from the 29 municipalities in the pilot project. The revision simply consisted of the addition of a comment field to each question, enabling the respondents to elaborate their answers, if necessary. When revision of the questionnaire was complete, all 98 municipalities were invited to respond to the questionnaire through the process described above. Informed consent for study participation was obtained.

### Demographics

Demographic data were gathered from the April 2017 census by Statistics Denmark, using the publicly available Statbank [19]. Data regarding the prevalence of citizens in each municipality originating in countries with tradition of FGM was gathered in order to compare the municipalities participating and not participating in the study, and to evaluate whether a high prevalence of citizens from risk countries was correlated with a higher possibility of having implemented a specific action plans against FGM.

First-generation immigrants are defined as individuals born in a different country than Denmark, and who have achieved status of permanent residents in Denmark at the time of the census. Second-generation immigrants are defined as individuals born in Denmark to two first-generation immigrant parents, and with a status of permanent residency in Denmark at the time of census. Risk countries were defined as countries with a tradition of FGM, and were identified in accordance with the UNICEF definition from 2016 [1], the Embará people of Colombia being excluded due to lack of data regarding the number of people from this ethnic group residing in Denmark. High-risk countries were defined as countries with FGM prevalence above 50%.

### Statistics

Quantitative data are presented as frequencies and proportions. Statistical analysis was performed using IBM SPSS Statistics (version 19 for iOS, IBM Corporation, Armonk, NY). Figures were created using GraphPad Prism (version 6 for iOS, GraphPad Software, San Diego, CA).

## Results

All 98 municipalities in Denmark were invited to participate in the study by responding to a questionnaire. A total of 67 municipalities participated in the study. The municipalities that did participate in the study had an average of 1.09% of the population with origins in countries with tradition of FGM and a median of 0.86%. The municipalities that did not participate in the study had an average of 1.21% and a median of 0.84%.

### Demographics

As of the first of April 2017, a total of 91,121 first- and second-generation immigrants with origins in countries with a tradition of FGM resided in Denmark. This equals 1.8% of Danish population. A total of 41,896 of these were females. A total of 33,198 first- and second-generation immigrants originated from the 12 high-risk countries, comprising 0.7% of the total Danish population. A total of 14,929 of these were females.

**Fig. 1:**
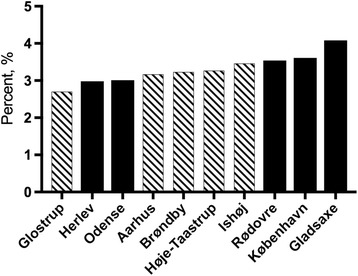
Prevalence of citizens with origins in risk countries in the ten highest-prevalence municipalities. Municipalities that did not respond to the questionnaire are indicated with shaded bars

The percentage of the population being first- or second-generation immigrants from countries with a tradition of FGM varied within the municipalities from 0% to 4.1% with a median of 0.8%. Only one municipality had 0% of the population originating in countries with a tradition of FGM. The ten municipalities with the highest prevalence of citizens with origins in risk countries were all situated within or in immediate proximity to the three most populated cities in Denmark (Fig. 1).

### Questionnaire

Firstly, the municipalities were asked whether they regard FGM as a current problem in their municipality. One (1.5%) municipality regarded FGM as a current problem within its respective municipality, 59 (88%) answered “no” and seven (10.4%) answered “do not know”. To the question of whether the municipality currently receives petitions concerning FGM from citizens and social workers, 47 (70.1%) municipalities answered “no”, seven (10.4%) municipalities answered “yes” and 13 (19.4%) answered “do not know”.

### Action plans

A total of seven (10.4%) municipalities stated that they had implemented a specific action plan for cases regarding FGM. Of these seven, one municipality, Copenhagen, made their action plan available upon request, while the remaining six municipalities did not. A total of 57 (85%) municipalities stated that they do not have a specific action plan, while three (4.5%) replied, “do not know”.

The municipality of Copenhagen was the only out of the aforementioned ten municipalities with the highest population share of citizens originating in a risk country, to state that they have implemented a specific action plan for combatting FGM.

Of the 57 municipalities where no specific action plan has been implemented, 13 (22.8%) municipalities indicated that FGM is included in other action plans within the given municipality. One municipality stated to be in the process of developing an action plan.

Thus, 46 municipalities (68.7%) indicated that they have not implemented a specific action plan regarding FGM, that FGM currently is not included in other action plans, that they are currently not in the process of developing a specific action plan towards cases of FGM or that they do not know whether an action plan has been implemented in their municipality.

### Preventive initiatives

Five (7.5%) municipalities stated that they have developed and implemented preventive initiatives to combat FGM. These municipalities defined their initiatives in the comment field sections as follows: 1) outreach conversations with parents and families in relevant groups; 2) lectures about Danish legislation regarding FGM during Danish language courses for immigrants; 3) preschool interviews with the school nurse, and 4) outreach healthcare visits.

A total of 44 (65.7%) municipalities replied “no” to having implemented preventive initiatives against FGM. Of these, one (2.3%) municipality stated to be in the process of preparing implementation of specific initiatives and preventive work against FGM, whereas 43 (97.7%) municipalities stated not to be. Eighteen (26.9%) municipalities answered “do not know”, to whether they had implemented preventive initiatives against FGM.

### Registration

A total of 49 (73.1%) municipalities indicated to have implemented routine registration of cases of FGM or suspicion hereof, whereas seven (10.5%) municipalities reported not to perform routine registration, and 11 (16.4%) were unaware if registration is performed. To the question of whether the municipalities register cases of FGM specifically and not under other categories of abuse or violence against children, 12 (17.9%) municipalities answered “yes”. A total of 22 (32.8%) municipalities stated to register cases of FGM under other categories concerning abuse or violence towards children. To the question of whether data from these registrations were available upon request, two (3%) municipalities answered “yes”. These two municipalities had unfortunately not made this data accessible to the public.

### Information material

Five (7.5%) municipalities indicated that they offer booklets or other written information material to the citizens or relevant professionals regarding FGM, while 54 (80.6%) municipalities stated that no information material of any kind had been developed or were available. Eight (11.9%) municipalities responded “do not know” to this question. Of the 10 municipalities with the highest share of citizens from risk countries, one municipality stated to offer information material about FGM to citizens and/or relevant professionals.

## Discussion

It was in the light of the fact that Denmark has not yet implemented a coordinated national action plan to combat FGM that we chose to undertake the present study to investigate whether the task of combating FGM was satisfactorily managed on the local level by the 98 Danish municipalities. Other Scandinavian countries have developed and implemented national action plans in order to create national as well as regional foundations for preventing FGM. In Sweden, FGM was prohibited in 1982, and several state prevention programmes was implemented from 1993. A national action plan was established in Sweden in 2003, as well as comprehensive guidelines from The Swedish Board of Health and Welfare to officials in the social sector. Johndottor et al. states that when dealing with cases of FGM, a crucial and facilitating factor is having guidelines so as to achieve knowledge of how to act practically when dealing with victims of FGM [13]. In Norway, a national action plan was developed and implemented in 2000 and a subsequent 3-year national project against FGM was launched in 2001 with continuous evaluations throughout the following years [16].

Our study showed that only 10.4% of the responding municipalities reported to have implemented a specific action plan to combat FGM. Our study also showed that only a small minority of Danish municipalities have implemented specific preventive efforts against FGM, have developed specific registration procedures for cases of FGM and/or have information material available for distribution. Furthermore, there seems to be no relation between the prevalence of immigrants with origins in risk countries and the probability of a certain municipality having implemented a specific action plan to combat FGM. Unfortunately, these facts are not in accordance with recommendations made by international actors in the field. It is recommended to implement a national action plan, and to combat and perform registration of FGM separately from other forms of abuse and violence towards children and adolescents [20–23]. A national action plan will furthermore ensure a consistent and uniform approach to the issue of FGM across municipality boundaries.

A total of seven municipalities reported to have received petitions regarding FGM cases; however, only one municipality stated that they view FGM as a current problem within the municipality. It can only be speculated whether the relatively rare occurrence of FGM petitions in Danish municipalities is a result of a general low incidence of FGM in Denmark, or is a result of a lack of coordinated registration and awareness among front workers.

In England, the introduction of coordinated registration systems in regards to cases of FGM revealed that over 1700 women and girls had undergone FGM and was treated by the NHS between in September 2014. This information was crucial so as to form a picture of the prevalence of FGM in the country and in order to support national prevention programmes [24]. There is currently no such national registration system in Denmark. Our study shows that registration of FGM specifically as such is performed in only 17.9% of Danish municipalities, and that 19.4% were unaware if there had been petitions received regarding FGM in their respective municipalities. These results suggest that Danish municipalities lack the necessary data to draw reliable conclusions as to whether or not FGM is a current problem.

A study by Van Baelen et al. based on demographic data from 2011 estimated that 7910 girls and women living in Denmark had been subjected to FGM before immigration [4]. The current prevalence of girls and women having been, or in risk of being, submitted to FGM in Denmark is unknown. A 2016 study by Ziyada et al. on FGM prevalence in Norway, which has a comparable number to Denmark of immigrants with origins in risk countries, estimated a prevalence of 17,300 girls and women having been subjected to FGM, and a prevalence of 3000–7900 girls in risk of being subjected to FGM [7]. We strongly recommend further investigation into the current prevalence of FGM in Denmark using the definitions, models and methodological approaches as recommended by the 2013 and 2015 EIGE reports [25, 26].

It is a weakness of the study that it does not offer explanations as to the reasons why the respective municipalities have chosen not to implement specific preventive measures or action plans against FGM. It is also a weakness that we did not gather data describing the responders of the questionnaire with regard to their knowledge about FGM, laws and their respective amount of influence within the respective municipalities. Furthermore, it was not possible to externally validate our data collected from the questionnaire, given the fact that there is no existing studies or data sources containing information enabling us to corroborate our findings. It could also be considered a weakness of our study that only 67 of the 98 municipalities responded to the questionnaire. In particular, of the ten municipalities in Denmark with the highest prevalence of immigrants with origins in risk countries, five municipalities did not participate in the study. However, the response rate of 68% is in line with what is considered a successful response rate for an online survey [18, 27–29]. The questionnaire consisted of quite few categories, which limited the extent of the results, but it could also be argued that this was a strength considering the relatively high response rate for an online survey.

## Conclusions

In conclusion, our study shows that the current situation of FGM registration and prevention being under local administrative responsibility, has led to a severe lack of coordinated public initiative against FGM in Denmark. Besides the lack of a coordinated, national action plan there seems to be a general lack of public awareness on the issue. In October 2017, the second conviction on FGM in Denmark was given to two parents for having their daughters subjected to FGM abroad. In the High Court of Western Denmark, the President of the court halved the sentence from the District Court from 18 to 9 months of prison, arguing “There has not been provided evidence of the procedure having consequences for the girls including consequences for their sexual health” [30]. This sentence was referred to in the national newspapers, but we have not been able to find any news articles questioning the sentence or the citation of the President of the High Court of Western Denmark, despite the fact that the international research on FGM has shown all types of FGM to be harmful practices, severely jeopardizing the health of women and girls [1, 31–35].

As such, Denmark does not live up to international recommendations in the field and it should be considered to implement nationally coordinated efforts against FGM such as an elaborate action plan, coordinated data collection and research programs to ensure a consistent approach towards FGM throughout the nation.

## Additional file

Additional file 1: Questionnaire. (DOCX 75 kb)

## Abbreviations

EIGE: European Institute for Gender Equality
FGM: Female genital mutilation
WHO: World Health Organization
