# Supplementary material for: Mapping the lack of public initiative against female genital mutilation in Denmark
Source: Reprod Health. 2018 Apr 7;15:59. doi: 10.1186/s12978-018-0499-2 (PMC5889535; doi:10.1186/s12978-018-0499-2)
Supplement: Supplementary file 1 — Questionnaire. (DOCX 75 kb) [file 12978_2018_499_MOESM1_ESM.docx]

**Appendix 1**

Questionnaire

**Prevention, registration and actions against female genital mutilation in Denmark**

According to the UN, female genital mutilation (FMG) is a violation of human rights, and the Children’s Convention commits nation states to perform preventive measures against FGM. This commitment is the background for our study, which aims to uncover the number of municipalities that have implemented specific action plans against FGM.

In addition, the study aims to illustrate the extent of the registration rate of FGM in Denmark.

Thank you for your help.

**OVERALL**

1. Do you consider female genital mutilation a widespread problem in your municipality?

Yes

No

Do not know

Comments:

|  |
| --- |

**ACTION PLAN**

2. Have you implemented a specific action plan for cases of female genital mutilation?

* An action plan in the terms of a point-by-point guideline for how professionals should act in case they become familiar with a case of female genital mutilation (or a girl at risk of being subjected to female genital mutilation).

Yes

No

Do not know

*If yes – proceed to question no. 6.*

Comments:

|  |
| --- |

3. Do you include the female genital mutilation area in other municipal actions plans? (eg action plans regarding physical/mental violence against children)

Yes

No

Do not know

*If yes – proceed to question no. 6.*

Comments:

|  |
| --- |

4. Are you in the process of developing a specific action plan for cases of female genital mutilation?

Yes

No

Do not know

*If yes – proceed to question no. 6.*

Comments:

|  |
| --- |

5. Are you in the process of incorporating an action plan for cases of female genital mutilation in another municipal action plan? (eg action plans regarding physical/mental violence against children)

Yes

No

Do not know

Comments:

|  |
| --- |

**REGISTRATION**

6. Do you receive reports concerning female genital mutilation in the municipality?

Yes

No

Do not know

Comments:

|  |
| --- |

7. In the case of female genital mutilation or suspicion hereof, do you register this?

Yes

No

Do not know

*If no – proceed to question 10.*

Comments:

|  |
| --- |

8. How do you register cases of female genital mutilation?

a. Specifically as such

b. Generally as physical violence towards children

c. Do not know

*If the above answer is not a. – then proceed to question 10.*

Comments:

|  |
| --- |

9. Do you have available numbers on these registrations? (Please state the number)

Yes

No

Do not know

Number:

|  |
| --- |

Comments:

|  |
| --- |

10. If professionals with contact with children (schoolteachers, healthcare professionals, educators, etc.) in your municipality acquire knowledge about a potential case of female genital mutilation, where/whom should they contact within your municipality?

|  |
| --- |

Comments:

|  |
| --- |

**INFORMATION**

11. Does your municipality provide a booklet or other relevant information material to hand out as preventive work against female genital mutilation?

Yes

No

Do not know

*If no – proceed to question nr. 14.*

Comments:

|  |
| --- |

12. In which language is this information available?

|  |
| --- |

Comments:

|  |
| --- |

13. Toward whom is this information material addressed?

a. Professionals

b. Children

c. Adults

d. Others

Comments:

|  |
| --- |

**PREVENTIVE INITIATIVES**

14. Are there other preventive efforts being carried out towards female genital mutilation in the municipality?

Yes

No

Do not know

*If no – proceed to question no. 16.*

Comments:

|  |
| --- |

15. Kindly describe these efforts.

|  |
| --- |

Comments:

|  |
| --- |

16. Is your municipality in the process of planning preventive efforts regarding female genital mutilation?

Yes

No

Do not know

Comments:

|  |
| --- |

17. Other remarks?

|  |
| --- |
